# T2-Sampling Perfection With Application-Optimized Contrasts by Using Flip Angle Evolution (SPACE) Protocol MRI: A Safe, Minimally Invasive Screening Tool for Spinal CSF Leak Causing Spontaneous Intracranial Hypotension

**DOI:** 10.7759/cureus.26626

**Published:** 2022-07-07

**Authors:** Bob Daripa, Scott Lucchese

**Affiliations:** 1 Internal Medicine/Neurology, Singapore General Hospital, Singapore, SGP; 2 Neurology, University Hospital, Missouri University Health Care, Columbia, USA; 3 Medicine, Grant Government Medical College and Sir J.J. (Jamsetjee Jeejeebhoy) Group of Hospitals, Mumbai, IND; 4 Neurology/Headache, University of Arkansas for Medical Sciences (UAMS), Little Rock, USA; 5 Neurology/Headache, University of Missouri School of Medicine, Columbia, USA

**Keywords:** screening tool, ds myelogram, spontaneous intracranial hypotension (sih), spinal csf leak, t2-space protocol mri

## Abstract

Spontaneous intracranial hypotension (SIH) due to a spinal cerebrospinal fluid (CSF) leak is secondary cause of headache with potentially devastating consequences. Its diagnosis is complicated owing to the lack of a reasonable, minimally invasive screening test. This results in many patients remaining undiagnosed for years after the headache onset. Current testing approaches are either overly invasive, namely the CSF infusion protocol or both invasive and insensitive viz. lumbar puncture (LP) with an opening pressure (OP) or computed tomography myelogram (CTM). These diagnostic methods are frequently employed in a clinical setting since they require access to the thecal space; they unfortunately have a dearth of sensitivity. CTM will not document a leak if it is intermittent or very slow and in the setting of a spinal CSF leak, the OP on LP may be high, low, or normal. A potential remedy for this state is the T2-sampling perfection with application-optimized contrasts by using flip angle evolution (SPACE) protocol spinal magnetic resonance imaging (MRI). We present two cases that demonstrate its potential value as a screening tool. It is well known for its high sensitivity for identifying spinal pathology and is minimally invasive, making it a good choice for a screening modality when diagnosing possible SIH cases.

## Introduction

While spontaneous intracranial hypotension (SIH) is an uncommon cause of intractable headache [1,2] it is commonly encountered due to a spinal cerebrospinal fluid (CSF) leak [1]. Spontaneous intracranial hypotension is associated with not only severe head pain but also other multiple disabling symptoms and findings including cognitive dysfunction, behavior changes, vision changes, diplopia, and many more [3]. Often SIH unveils as a subtle syndrome and may go undiagnosed for years. Recognizing this entity requires clinical skills, but even in the presence of excellent history and physical examination, the diagnosis may get delayed because of the lack of a reasonable screening test. Current testing is either overly invasive namely CSF infusion protocols or both invasive and insensitive such as a lumbar puncture (LP) with an opening pressure (OP) or computed tomography myelogram (CTM) [2]. The later two procedures are commonly employed in the diagnostic workup and require access to thecal space; they unfortunately lack sensitivity. Computed tomography myelogram will not identify a leak if it is intermittent or very slow and in the setting of a spinal CSF leak the opening pressure on LP may be high, low, or normal [2]. Recent studies concluded that the T2-sampling perfection with application-optimized contrasts by using flip angle evolution (SPACE) is superior to the T2 fast spin echo (FSE) MR imaging for visualizing the cervical spine as it has fewer CSF flow/pulsation artifacts the latter of which ameliorate visibility [4]. We present two cases that presented with a consistent history of headaches suggestive of SIH. The Bern score for each was ≥5 implying the high probability of a spinal leak [5]. The T2-SPACE MRI spine helped visualize the CSF leaks in these cases which were later confirmed by digital subtraction myelogram (DSM).

The abstract of this article was previously presented as an e-poster at the International Headache Congress (co-organized by the International Headache Society and European Headache Federation), held virtually in Berlin, Germany, on September 8-12, 2021 (Daripa B, Lucchese S: T2-SPACE protocol MRI: a safe, minimally invasive screening tool for spinal CSF leak causing spontaneous intracranial hypotension {P0253}. J Headache Pain. 2021, 22:1-153).

## Case presentation

Case 1

**Figure 1:**
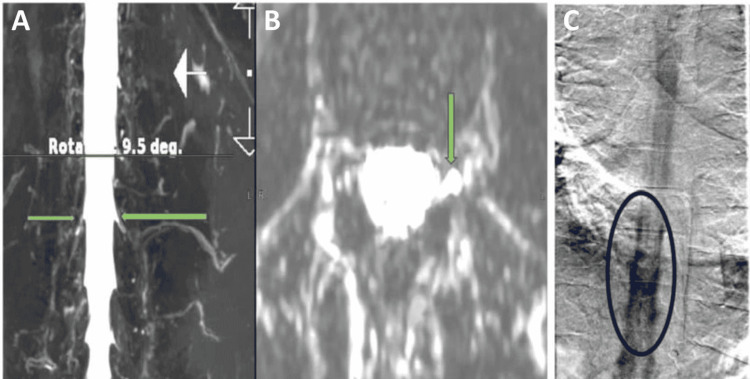
The thoracolumbar longitudinal and cross-sectional T2-SPACE MR myelogram images. (A and B) At the T11-T12 level, green arrows indicate fluid outside thecal space associated with a possible CSF leak. (C) DS myelogram confirms the localization of the CSF leak. Dye escaping the thecal sac can be observed at the level T12 to L1 (blue oval circle). The image is taken from Daripa B, Lucchese S: T2-SPACE protocol MRI: a safe, minimally invasive screening tool for spinal CSF leak causing spontaneous intracranial hypotension (P0253). J Headache Pain. 2021, 22:1-153. MR: magnetic resonance; CSF: cerebrospinal fluid; DS: digital subtraction

A 32-year-old thin female with no comorbidities presented to the neurology clinic complaining of a headache. It was a chronic disabling headache for which she had tried various medications with minimal benefit. The headache was holocranial, prominent in the occipital region, and exhibited a postural variation associated with throbbing upon standing. There were no associated ophthalmological complaints during the headache attacks nor other related warning symptoms or signs. She does have phonophotophobia during her attacks and the initial clinical characteristics were supportive of migrainous headaches. The patient's headache was relieved by sleep, but as the day progressed, it grew worse. She claims that she is good at performing stretching exercises and regularly does yoga. On examination, her body mass index (BMI) was 18 kg/m^2^. Her vital signs were normal with no significant findings on neurological examination. The routine blood workup including the vascular and autoimmune panels was normal. The CSF OP was 8 cm of H_2_O and the CSF analysis was normal. The clinical symptomatology along with the findings of the MRI brain scan was suggestive of SIH and with a BERN score of 6, implying the high probability of the spinal leak [5]. On spinal MRI imaging, we found excessive fluid signal in an area that was typical for the development of a CSF leak site, i.e., at the T11-T12 levels [6]. The T2-SPACE MRI spine helped visualize the CSF leak which was later confirmed by the lateral decubitus digital subtraction myelogram (DSM) (Figure 1).

Case 2

**Figure 2:**
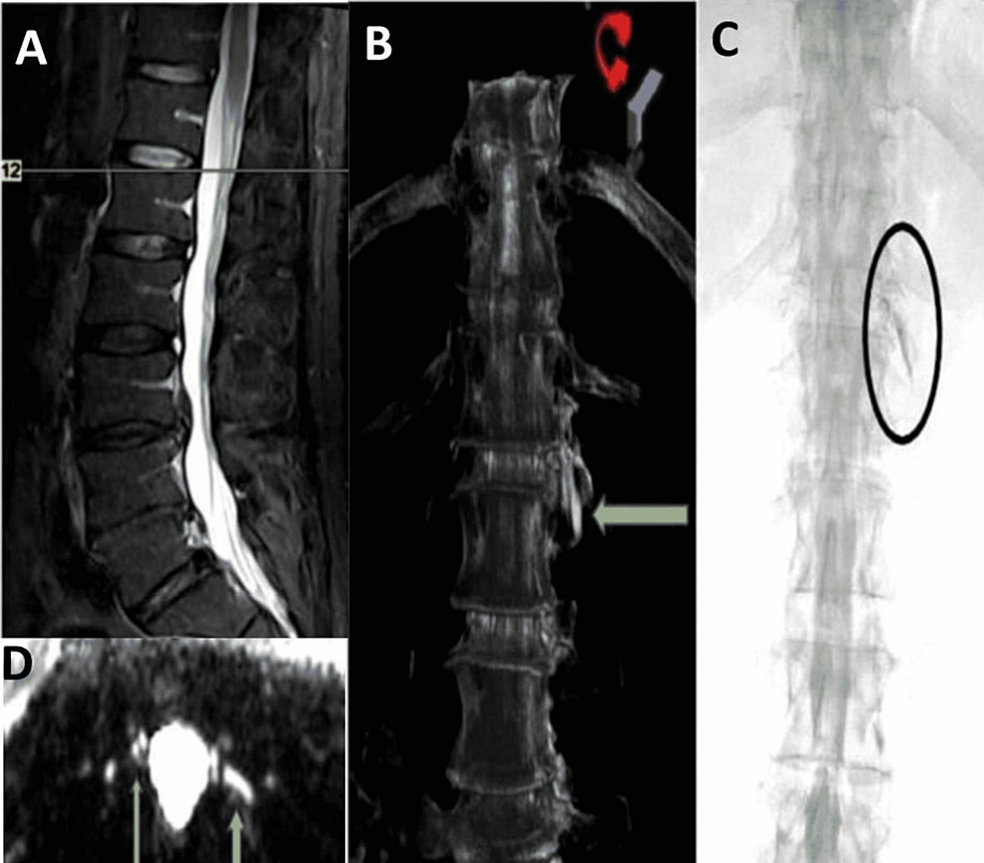
The thoracolumbar sagittal and cross-sectional T2 MR myelogram imaging. (A and D) At the T12 level, green arrows indicate an area of fluid outside the thecal space. (C) The DS myelogram image shows dye extravasation visualized in the black oval ring. (B) A 3D-reconstruction image illustrates the leak site localization at T12 (thick green arrow). The image is taken from Daripa B, Lucchese S: T2-SPACE protocol MRI: a safe, minimally invasive screening tool for spinal CSF leak causing spontaneous intracranial hypotension (P0253). J Headache Pain. 2021, 22:1-153. MR: magnetic resonance; CSF: cerebrospinal fluid; DS: digital substraction

A 45-year-old, middle-aged, healthy, and muscular male presented with a history of chronic headaches. It was a unilateral to holocranial headache with shifting laterality incorporating features of both migraine and tension-type headaches. This headache also had a postural component where lying helped the headache but the degree of relief was inconsistent. The headache intensity was gradually progressive in the last six months. He recalled a plane flight a few months back that precipitated a holocranial head heaviness. An LP procedure done at that time was reported as normal. The CSF OP was 16 cm of H_2_O with a normal cell analysis. Clinically, we considered the possibility of SIH after identifying the cranial findings of SIH in each patient's brain MRI scan and Bern score of six [5]. A recent T2-SPACE spinal MR myelogram imaging suggested the presence of CSF fluid outside the thecal space at the level of T12, which was well illustrated in Figure 2 and was further supported by a 3D-reconstruction image localizing the leak site. The DS myelogram confirmed the dye extravasation at the same level.

## Discussion

The identification of a probable CSF leak site is relatively easy using the T2-SPACE protocol MRI. T2-SPACE MRI can be utilized as a screening modality for ascertaining a spinal CSF leak and also for monitoring a post-blood patch seal. The identification of excess fluid signal outside the thecal sac aids in deciding the need for additional investigation to determine the localization of the leak. An abnormal fluid collection is not specific for a CSF leak but the sensitivity seems sufficient to be considered as a screen test. In the setting of probable CSF pooling seen on a T2-SPACE protocol MRI, it would seem reasonable to proceed with the more specific testing viz. DSM or dynamic computed tomography myelography (DCTM) post-MRI [7]. This approach should help decrease the latency from disease onset to diagnosis as it is more likely to be ordered in the setting of lesser suspicion than perhaps is required for a physician to justify an invasive CT myelogram.

A DSM is exhaustive and uncomfortable. Its invasive nature and radiation exposure are also less than ideal as a screening test, though it is both sensitive and specific. It conveys two to four fold more radiation than conventional fluoroscopy. Digital subtraction myelography is sensitive to breathing and motion artifacts altering image quality [1]. Both DSM and conventional dynamic myelography (CDM) [7] are limited to 2D images and cannot generate cross-sectional images [1]. Dynamic computed tomography myelography (DCTM) is also associated with a high radiation dose and can cover only a few vertebral levels. The bony structures may also obscure the leak site [7].

## Conclusions

T2-SPACE protocol spinal MRI has a potential value as a screening tool in identifying a spinal CSF leak. It is well known for its high sensitivity to spinal pathology and is minimally invasive, making it a good choice for screening modality when evaluating possible SIH cases. More sensitive, specific, and definitive testing can be employed post-T2-SPACE MRI, as they have the capability to ascertain the leak's location in a shorter time along with reduced radiation exposure for targeted intervention.
